# Anti-Inflammatory Effects of *Hyeonggaeyeongyo-tang*: Evidence from In Vitro and In Vivo Studies

**DOI:** 10.3390/life15040587

**Published:** 2025-04-02

**Authors:** Keun Hee Lee, Min Hee Kim, Hae Jeong Nam

**Affiliations:** 1 Department of Clinical Korean Medicine, Graduate School, Kyung Hee University, Seoul 02447, Republic of Korea; lkh752@nate.com; 2 Kapsan Oriental Medicine Clinic, Gyeongju 38203, Republic of Korea; 3 Department of Ophthalmology, Otolaryngology, and Dermatology, Kyung Hee University College of Korean Medicine, Kyung Hee University Hospital at Gangdong, Seoul 05278, Republic of Korea; minhee@khu.ac.kr; 4 Department of Ophthalmology, Otolaryngology, and Dermatology, Kyung Hee University College of Korean Medicine, Kyung Hee University Medical Center, Seoul 02447, Republic of Korea

**Keywords:** hyeonggaeyeongyo-tang, anti-inflammatory, histopathology, rat paw edema, RAW 264.7

## Abstract

*Hyeonggaeyeongyo-tang* (HGYGT), a traditional herbal formula, is used to treat inflammatory otorhinolaryngological diseases such as otitis media and sinusitis. In this study, we investigated the anti-inflammatory effects of HGYGT in LPS-stimulated RAW 264.7 cells (in vitro) and a carrageenan (CA)-induced rat paw edema model (in vivo). In LPS-stimulated RAW 264.7 cells, treatment with HGYGT (100 and 300 μg/mL) significantly reduced nitric oxide (NO) production by 24.5% and 51.3%, respectively (*p* < 0.05, *p* < 0.01). It also significantly suppressed the production of PGE2 (49.8%), IL-1β (42.7%), IL-6 (45.6%), and TNF-α (47.2%) at 300 μg/mL (*p* < 0.01). A Western blot analysis confirmed that HGYGT (300 μg/mL) significantly downregulated iNOS and COX-2 expression by 58.4% and 53.1%, respectively, while COX-1 remained unaffected. And HGYGT treatment at 300 μg/mL markedly inhibited NF-κB activation by 44.9% (*p* < 0.01). Furthermore, HGYGT selectively inhibited JNK phosphorylation by 46.7% (*p* < 0.01), without significantly affecting ERK1/2 or p38 MAPKs. In the CA-induced rat paw edema model, oral administration of HGYGT (1.0 g/kg) reduced paw swelling by 31.5% at 4 h post-injection (*p* < 0.01) and significantly decreased iNOS expression in inflamed paw tissues by 43.2% (*p* < 0.01). A histological analysis revealed that HGYGT (1.0 g/kg) reduced inflammatory cell infiltration by 39.6% in the affected tissue (*p* < 0.05), demonstrating its anti-inflammatory potential. Our findings demonstrate that HGYGT exerts anti-inflammatory effects by suppressing the JNK and NF-κB signaling pathways in LPS-stimulated RAW 264.7 cells, reducing the production of inflammatory mediators. Notably, HGYGT selectively inhibits COX-2 without affecting COX-1 and preferentially suppresses the JNK pathway. Moreover, its in vivo anti-inflammatory effects were confirmed through iNOS inhibition and histopathological analysis. These findings provide robust scientific evidence supporting the traditional use of HGYGT and its anti-inflammatory properties.

## 1. Introduction

*Hyeonggaeyeongyo-tang* (*Jinglielianqiaotang*, HGYGT) is a traditional Chinese medicine (TCM) herbal prescription used to treat various disorders caused by wind-heat rising [1]. In TCM, wind heat refers to a pathological condition where excessive heat and external wind disrupt the body’s balance, often leading to inflammatory symptoms such as fever, redness, swelling, sore throat, and skin rashes. This concept aligns with modern understandings of inflammatory responses. Therefore, HGYGT is a representative prescription for inflammatory diseases and has been used for various ear, nose, and skin conditions with only a few differentiated herbal ingredients.

HGYGT includes the following common herbal ingredients: *Schizonepeta tenuifolia* Briq, *Bupleurum falcatum* L., *Cnidium officinale* Makino, *Angelica gigas* Nakai, *Paeonia lactiflora* Pall, *A. dahurica* (Hoffm.) Benth. & Hook.f. ex Franch. and Sav, *Saposhnikovia divaricata* (Turcz.) Schischk, *Gardenia jasminoides* J. Ellis, *Scutellaria baicalensis* Georgi, *Platycodon grandiflorum* (Jacq.) A.DC, *Forsythia suspensa* (Thunb.) Vahl, and *Glycyrrhiza uralensis* Fisch. However, in some cases, *Citrus aurantium* L. was added, whereas in other instances, *Rehmannia glutinosa* (Gaertn.) DC and *Mentha arvensis* L. were added.

Previous studies investigated the pharmacological effects of HGYGT in various experimental research studies. HGYGT has been shown to exhibit antipyretic, analgesic, and anti-inflammatory properties [2]. It has also demonstrated anti-allergic effects in animal models [3] and anti-inflammatory, analgesic, and anti-allergic activities in separate studies [4]. Kim et al. [5] reported that HGYGT suppressed lipopolysaccharide (LPS)-induced nitric oxide (NO) production, cytokine release, and the expression of inducible nitric oxide synthase (iNOS) and cyclooxygenase-2 (COX-2) in cellular models. Additionally, HGYGT demonstrated immunomodulatory effects in animal models of atopic dermatitis [6,7,8,9]. Its inhibitory effects on iNOS expression have also been observed in mouse cells [10] and mice with allergic rhinitis [11]. HGYGT was effective in a mouse model of ovalbumin-induced allergic rhinitis [12]. HGYGT’s antibacterial, antioxidant, and anti-inflammatory properties have also been confirmed in cellular studies [13]. However, most studies on HGYGT have been conducted on the formulation with *C. aurantium* L. added, for some reason.

*R. glutinosa* exhibits various pharmacological effects, including antioxidant, anti-inflammatory, immunomodulatory, neuroprotective, bone metabolism regulatory, antidepressant, anti-diabetic, cardiovascular, liver, kidney, and lung function improvements, as well as antitumor and anti-aging properties [14]. *M. arvensis* has demonstrated antioxidant, antibacterial, antifungal, anti-yeast, antiviral, and antitumor activities [15]. Thus, it can be anticipated that HGYGT with added *R. glutinosa* (Gaertn.) DC and *M. arvensis* L. will exhibit a significant anti-inflammatory effect.

Therefore, in this study, we conducted experiments using HGYGT with the addition of *R. glutinosa* (Gaertn.) DC and *M. arvensis* L. In this study, the anti-inflammatory effects of HGYGT were evaluated in vitro and in vivo. In vitro, in addition to previously investigated factors such as NO production, cell viability, iNOS, COX-2, and cytokines, including interleukin-1 beta (IL-1β), interleukin-6 (IL-6), and tumor necrosis factor-alpha (TNF-α), further analyses were conducted on prostaglandin E2 (PGE2), cyclooxygenase-1 (COX-1), inhibitor of kappa B alpha (I-κBα), nuclear factor kappa light-chain enhancer of activated B cells (NF-κB), and mitogen-activated protein kinases (MAPKs), including c-Jun N-terminal kinase (JNK), extracellular signal-regulated kinase 1/2 (ERK1/2), and p38 MAPK. In vivo, along with the previously assessed carrageenan (CA)-induced paw edema volume and liver function markers aspartate aminotransferase (AST) and alanine aminotransferase (ALT), additional evaluations included iNOS expression and histological examinations to comprehensively investigate the anti-inflammatory effects of HGYGT.

To highlight the distinctiveness of this study compared with previous research, we employed UPLC to quantify the standardized HGYGT extract, ensuring reproducibility. In the in vivo experiments, we also investigated the expression of COX-1 and PGE2, as well as the MAPKs pathway. In the in vitro experiments, we conducted further analyses of the histological morphology and iNOS expression to comprehensively elucidate the anti-inflammatory effects of HGYGT. Through this approach, we aimed to present a more advanced and integrated methodology than previous studies.

## 2. Materials and Methods

### 2.1. Chemicals and Reagents

To standardize the quality of HGYGT used in this study, some standard compounds were selected from the medicinal ingredients that constitute HGYGT. Forsythoside B from *F. suspensa*, decursinol from *A. gigas*, ligustrazine hydrochloride from *C. officinale*, paeoniflorin and paeonol from *P. lactiflora*, catalpol from *R. glutinosa*, berberine from *Scutellaria baicalensis*, geniposide from *G. jasminoides*, isoimperatorin from *A. dahurica*, platycodin D from *P. grandiflorum*, luteolin-7-glucopyranoside from *Schizonepeta tenuifolia* Briq, saikosaponin D from *B. falcatum* L., peucedanol from *Saposhnikovia divaricata* (Turcz.) Schischk, and glycyrrhizic acid from *G. uralensis* were selected as indicators and analyzed. Among the standards used in the experiment, forsythoside B, berberine, decursinol, platycodin D, ligustrazine hydrochloride, catalpol, geniposide, luteolin-7-glucopyranoside, saikosaponin D, peucedanol, and isoimperatorin were purchased from ChemFaces Inc. (Wuhan, China), paeoniflorin was purchased from Sigma-Aldrich (Merck KGaA, Darmstadt, Germany), and paeonol and glycyrrhizic acid were purchased from Wako Inc. (Osaka, Japan). Acetonitrile, methanol, and other solvents for ultraperformance liquid chromatography (UPLC) were purchased from J. T. Baker (Avantor, Center Valley, PA, USA).

Anti-iNOS (No. 610431) was purchased from BD Bioscience (San Jose, CA, USA), and anti-β-Actin (A5316) was purchased from Sigma-Aldrich (St. Louis, MO, USA). Anti-COX-2 (No. 160106) and anti-COX-1 (No. 160109) antibodies were purchased from Cayman Chemical Co. (Ann Arbor, MI, USA). Anti-IκBα (sc-371) was purchased from Santa Cruz Biotechnology (Santa Cruz, CA, USA). Anti-p-IκBα (#2859), anti-NF-κB (#8242), anti-Lamin A/C (#2032), anti-p-JNK (#9251), anti-JNK (#9252), anti-p-ERK (#9101), anti-ERK (#9102), anti-p-p38 (#9211), and anti-p38 (#9212) were purchased from Cell Signaling Technology (Danvers, MA, USA). Enzyme-linked immunosorbent assay (ELISA) kits for PGE2 (No. KGE004B) were purchased from R&D Systems (Minneapolis, MN, USA), and ELISA kits for IL-1β (No. BMS6002), IL-6 (No. KMC0061), and TNF-α (No. BMS607-3) were purchased from Thermo Fisher Scientific Inc. (Rockford, IL, USA). LPS, sodium nitrite, 3-(4,5-Dimethylthiazol-2-yl)-2,5-diphenyltetrazolium bromide (MTT), dexamethasone (DEXA), CA, and other chemicals were purchased from Sigma-Aldrich (St. Louis, MO, USA).

### 2.2. Preparation of HGYGT

HGYGT is composed of *Schizonepeta tenuifolia*, *B. falcatum*, *C. officinale*, *A. gigas*, *R. glutinosa*, *P. lactiflora*, *A. dahurica*, *Saposhnikovia divaricata*, *M. arvensis*, *G. jasminoides*, *Scutellaria baicalensis*, *P. grandiflorum*, and *F. suspensa*, (1.875 g [5 servings] each), along with *G. uralensis* (1.125 g [3 servings]) (Table 1). Herbs were purchased from Daewon Pharmaceutical Co., Ltd. (Daegu, Republic of Korea). HGYGT (102 g) was added to 1.5 L of water and extracted for 3 h in a Daewoong boiling pot at a ratio of approximately 1:14.7 (HGYGT–water), ensuring a consistency with traditional decoction methods. Among the constituent herbs, *Schizonepeta tenuifolia* and *M. arvensis* were added last and extracted for 20 min. The extract was filtered through a No. 2 filter paper (300 mm, Advantec, Tokyo, Japan) and concentrated using a rotary evaporator (BÜCHI Labortechnik AG, Flawil, Switzerland). The concentrate was frozen and freeze-dried using a freeze-dryer (Operon, Gimpo, Republic of Korea) to obtain the freeze-dried HGYGT products (yield: 27.89%). Freeze-dried HGYGT powder was dissolved in distilled water immediately before use and sterilized by filtration using a 0.2 µm syringe filter (Nalgene, Rochester, NY, USA) to prevent contamination.

### 2.3. Chemical Profiling of HGYGT by UPLC

#### 2.3.1. Chromatographic Conditions

A UPLC operating system was equipped with a pump (ACQUITY™ UPLC System, Waters Corporation, Milford, MA, USA) and a Waters ACQUITY^TM^ photodiode array (PDA) detector. The signal was detected using the Empower Data System. Separation was performed using a Waters ACQUITY^TM^ BEH C_18_ column [1.7 μm and 2.1 × 100]. The mobile phase consisted of 0.1% formic acid in acetonitrile and 0.1% formic acid in water with gradient elution (0.4 mL/min). The volume for injection was always 2 μL (Table 2). The analytical wavelength of PDA was 208 nm for catalpol; 210 nm for Saikosaponin D and Peucedanol; 254 nm for geniposide, glycyrrhizic acid, and isoimperatorin; 260 nm for ligustrazine hydrochloride; 275 nm for paeonol; 280 nm for forsythoside B, platycodin D and Luteolin-7-glucopyranoside; 330 nm for decursinol; 345 nm for paeoniflorin; and 380 nm for berberine, and the column temperature was set at room temperature.

#### 2.3.2. Preparation of Sample and Standard Solutions

To measure the content of each component corresponding to the standard product in the HGYGT extract, 0.2 g of homogeneously mixed freeze-dried HGYGT sample was taken, 10 mL of 70% methanol were added, and ultrasonic extraction was performed for 1 h. The extract was filtered through a 0.22 μm filter and used as a test solution. In addition, standard eleven solutions were prepared (1000 μg/mL [methanol]). All solutions were stored at 4 °C.

### 2.4. Cell Culture

The Research Applications of Wistar Institute (RAW) 264.7 mouse macrophage cell line (American Type Culture Collection, Manassas, VA, USA) were maintained in Dulbecco’s modified Eagle’s medium (DMEM; Hyclone, Thermo Fisher Scientific Inc., Logan, UT, USA) supplemented with 10% heat-inactivated fetal bovine serum (FBS; Sigma-Aldrich; Merck KGaA, Darmstadt, Germany), 100 U/mL of penicillin, and 100 µg/mL of streptomycin (Gibco, Thermo Fisher Scientific, Inc, Waltham, MA, USA) at 37 °C in a 5% CO_2_ incubator.

### 2.5. MTT Assay for Cell Viability

To determine the cytotoxic concentrations of HGYGT, RAW 264.7 cells were plated in a 96-well plate at a density of 5 × 10^4^ cells per well. The cells were serum-starved for 16 h, followed by pretreatment with a variety of concentrations of HGYGT for 1 h, then followed by stimulation with 1 µg/mL of LPS. The cells were then incubated for the next 20 h at 37 °C in a 5% CO_2_ incubator. Following incubation of the cells, viable cells were stained with 3-(4,5-dimethylthiazol-2-yl)-2,5-diphenyltetrazolium bromide (0.5 mg/mL, 4 h). The media were then removed, and formazan crystals produced in the wells were dissolved by the addition of 200 µL dimethylsulfoxide. The absorbance was measured at 570 nm using an ELISA microplate reader (Infinite 200 Pro; Tecan, Männedorf, Switzerland). Cell viability was calculated relative to that of untreated control cells using the formula Viability (% control) = 100 × (absorbance of treated sample)/(absorbance of control).

### 2.6. Measurement of NO

According to previously established procedures [16,17,18,19], RAW 264.7 cells (5 × 10^5^ cells/mL) were cultured for approximately 16 h and then treated with various concentrations of HGYGT for 1 h. Then, the inflammatory responses of the RAW 264.7 cells were induced with LPS (1 µg/mL). The cells were further cultured for 20 h in a 5% CO_2_ incubator (37 °C), and the culture supernatant was collected. NO was measured by reacting 100 µL of Griess reagent (0.1% N-(1-naphthy)-ethylenediamine dihydrochloride + 1% sulfanilamide in 5% phosphoric acid; Hoffmann-La Roche AG, Basel, Switzerland with 100 µL of the cell supernatant at 25 °C for 15 min. Absorbance was measured at 540 nm using a microplate reader (Tecan Group Ltd., Männedorf, Switzerland).

### 2.7. IL-1β, IL-6, TNF-α, and PGE2 Assays

RAW 264.7 cells were cultured at 5 × 10^5^ cells/mL for 16 h, then pretreated with various concentrations of HGYGT for 1 h. Then, they were stimulated with 1 µg/mL LPS. Twenty hours after LPS stimulation, the culture supernatants were collected, and the levels of IL-1β, IL-6, TNF-α, and PGE2 were quantified using a microplate reader (Tecan).

### 2.8. Preparation of Whole-Cell Extracts and Nuclear Fractions and Immunoblot Analysis

Whole-cell extracts were collected in a microtube with phosphate-buffered saline (PBS) after the cells were treated according to the goal of each experiment and centrifuged at 3000× *g* in a 4 °C centrifuge to leave only the cells in the microtube. One-hundred µL of lysis buffer containing radioimmnoprecipitation buffer and Halt protease and phosphatase inhibitor cocktail (Thermo Fisher Scientific Inc.) was added and vortexed, and the cells were reacted at 4 °C for 1 h. This was centrifuged again at 15,000× *g* for 10 min in a 4 °C centrifuge to obtain the supernatant, which was called the whole-cell extract (whole-cell lysate). Nuclear fraction lysates for protein evaluation were extracted from the treated cells using NER-PERTM Nuclear and Cytoplasmic Extraction Reagents (Thermo Fisher Scientific Inc.) according to the manufacturer’s instructions. The cell extracts were stored at −70 °C until the experiment. The protein of each extract was quantified using a bicinchoninic acid (BCA) kit, and the same volume of extract was electrophoresed by sodium dodecyl sulfate–polyacrylamide gel electrophoresis (SDS-PAGE). The protein was transferred to a nitrocellulose (NC) membrane and then reacted sequentially with primary and secondary antibodies, and the expression of each protein was visualized using an enhanced chemiluminescence (ECL)^®^ chemiluminescence detection kit (GE Healthcare Life Sciences, Buckinghamshire, UK). The protein expression level was quantified using an image analysis system (Amersham Imager 600), and the expression level of each protein was expressed as a multiple of that of each group compared with the control, 2nd ed. Beijing: Renminweisheng.

### 2.9. CA-Induced Paw Edema

Paw edema experiments were performed according to previously established procedures [16,17,18,19]. All procedures were conducted in accordance with the national regulations pertaining to the welfare and use of laboratory animals and were approved by the Institutional Animal Care and Use Committee of Daegu Haany University (Approval No. DHU2024-043). Male Sprague–Dawley rats (4 weeks old; weighing 80–100 g) were purchased from Samtako Co., (Osan, Republic of Korea) and acclimatized for 1 week. The animals were housed in a pathogen-free environment at a temperature of 20–23 °C, a 12 h light/dark cycle, and 50% relative humidity, with free access to commercial food (Nestle Purina PetCare Ltd., Seoul, Republic of Korea) and water. The rats (n = 25) were randomly assigned to five groups of five animals each. HGYGT was orally administered to the rats at doses of 0.3 and 1.0 g/kg/day for 3 consecutive days. DEXA, an anti-inflammatory drug, was used as the positive control. To induce acute inflammation, rats were subcutaneously injected with CA solution (1% in saline; 0.1 mL/rat) into the right hind paw 1 h after HGYGT treatment. Paw volumes were measured at 1 h intervals for 4 h after injection using a plethysmometer (Ugo Basile, Varese, Italy).

### 2.10. Histological Evaluation

The *Dorsum* and *ventrum pedis* skin of the hind paws was separated and fixed with 10% formalin, embedded in paraffin, sectioned (3–4 μm), and stained with hematoxylin and eosin (H and E). To observe the changes induced by CA treatment, the thicknesses of the *dorsum* and *ventrum pedis* skin were measured using an automated image analyzer (DMI-300; DMI, Daegu, Republic of Korea) under a microscope at 40× magnification (Nikon, Tokyo, Japan). The inflammatory cells infiltrating the dermis were counted (cells/mm^2^) at 200× magnification.

### 2.11. Blood Biochemistry

Plasma was separated from the blood samples, and the ALT and AST levels were examined using an automated blood analyzer (FUJI DRI-CHEMNX500I, Fuji Medical Systems Co., Ltd., Tokyo, Japan).

### 2.12. Statistical Analysis

All key experiments were independently repeated at least three times (biological replicates) to ensure reproducibility and statistical validity, with each experiment including technical triplicates. Statistical analysis was performed using SPSS version 26.0 (IBM Corp., Armonk, NY, USA). Statistical significance between groups was tested using a one-way analysis of variance. Post hoc tests were performed using Tukey’s honestly significant difference test (Tukey HSD test) or the Dunnett T3 test, and the results were expressed as mean ± S.D. Statistically, *p* values < 0.05 were rated significant. This approach ensures that the observed effects are consistent and not due to random variation.

## 3. Results

### 3.1. Analysis of HGYGT

**Figure 1:**
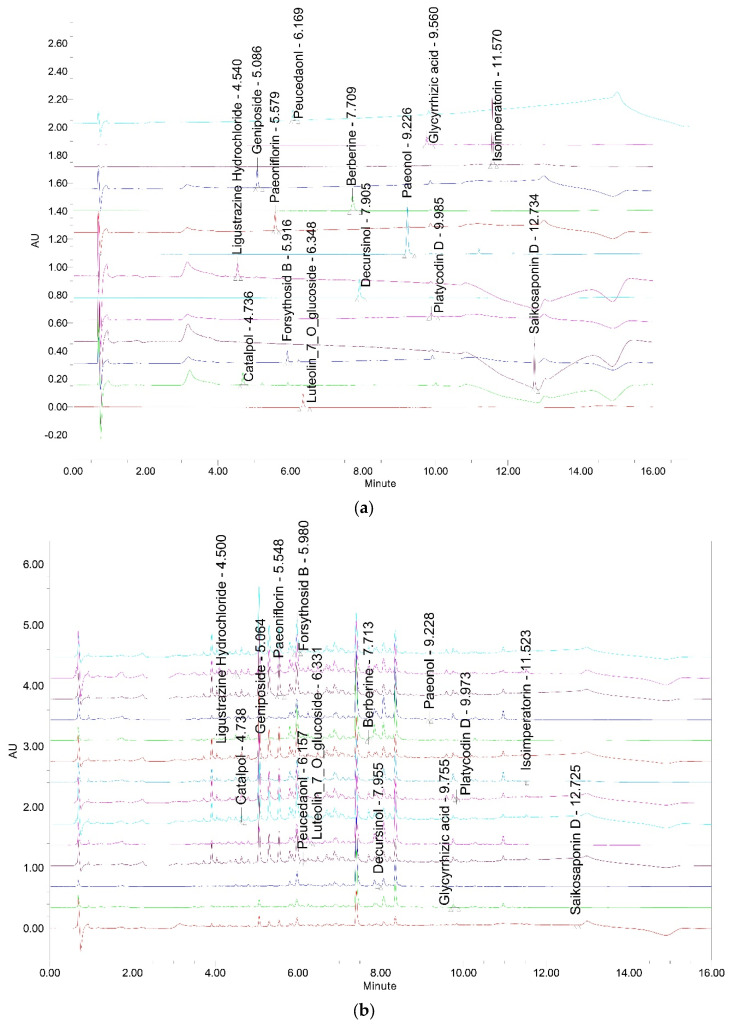
UPLC chromatogram of 14 marker compounds in HGYGT. (**a**) UPLC chromatogram of commercial standard compounds; (**b**) UPLC chromatogram of 14 marker compounds in HGYGT. The chromatograms were obtained at 208 nm (for catalpol), 210 nm (for saikosaponin D and peucedanol), 254 nm (for geniposide, glycyrrhizic acid, and isoimperatorin), 260 nm (for ligustrazine hydrochloride), 275 nm (for paeonol), 280 nm (for forsythoside B, platycodin D and luteolin-7-glucopyranoside), 330 nm (for decursinol), 345 nm (for paeoniflorin), and 380 nm (for berberine).

The determination of 14 markers, namely forsythoside B, platycodin D, ligustrazine hydrochloride, paeoniflorin, paeonol, catalpol, berberine, decursinol, glycyrrhizic acid, isoimperatorin, geniposide, luteolin-7-glucopyranoside, saikosaponin D, peucedanol, in HGYGT was established using the UPLC system. The concentrations of the 14 marker components were calculated from the calibration curve of the standards (Table 3 and Figure 1). The reliability and stability of the method were verified. This method resulted in the successive separation of 11 marker components in the HGYGT samples.

### 3.2. Effects of HGYGT on LPS-Stimulated NO Production and Cell Viability

**Figure 2:**
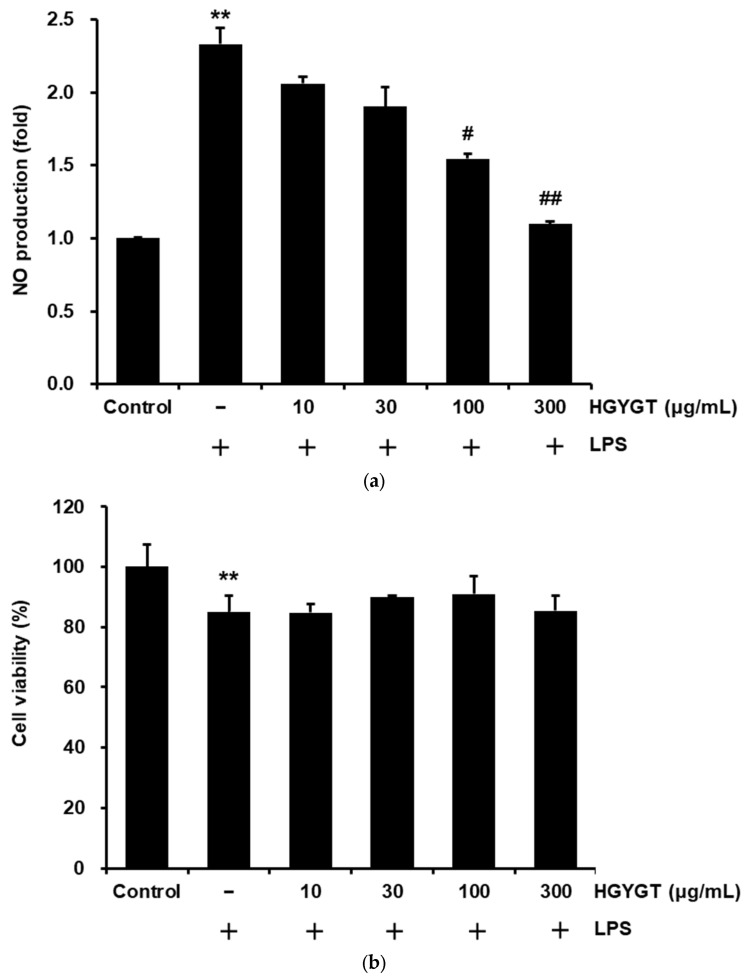
Effects of HGYGT on (**a**) nitric oxide (NO) production and (**b**) cell viability by lipopolysaccharide (LPS) in Research Applications of Wistar Institute (RAW) 264.7 cells. The cells at a concentration of 5 × 10^5^ cells/mL were treated with different concentrations (10–300 μg/mL) of HGYGT for 1 h, followed by induction with 1 µg/mL of LPS, and these cells were then incubated for 20 h. Control cells were incubated with vehicle alone. The concentrations of NO in the culture supernatant were recorded. In addition, the cytotoxic effects of HGYGT in the cells were determined by 3-(4,5-Dimethylthiazol-2-yl)-2,5-diphenyltetrazolium bromide (MTT) assay. Data are presented as mean ± S.D. of three replicates for each sample. Statistical analysis was performed using one-way analysis of variance (ANOVA), followed by Tukey’s honestly significant difference (HSD) or Dunnett T3 test. ** *p* < 0.01 significant compared with vehicle-treated control. ^#^ *p* < 0.05, ^##^ *p* < 0.01 significant compared with LPS only group.

To measure the inhibitory effects of HGYGT on LPS-stimulated NO production in RAW 264.7 cells, HGYGTs (10–300 μg/mL) were analyzed. When compared to the control, treatment with LPS (1 µg/mL for 20 h) significantly increased NO production (*p* < 0.01, Figure 2a). However, treatment with HGYGT (100 and 300 μg/mL) significantly reduced LPS-stimulated NO production by 33.8% and 52.9% (*p* < 0.05 for 100 and *p* < 0.01 for 300 μg/mL, Figure 2a). In addition, the possible cytotoxic effects of HGYGT on RAW 264.7 cells were examined using an MTT assay. When compared to the LPS only, the cell viabilities were not affected by treatment with HGYGT, at least up to a HGYGT concentration of 300 μg/mL (Figure 2b).

### 3.3. Effects of HGYGT on LPS-Induced PGE2 Production

**Figure 3:**
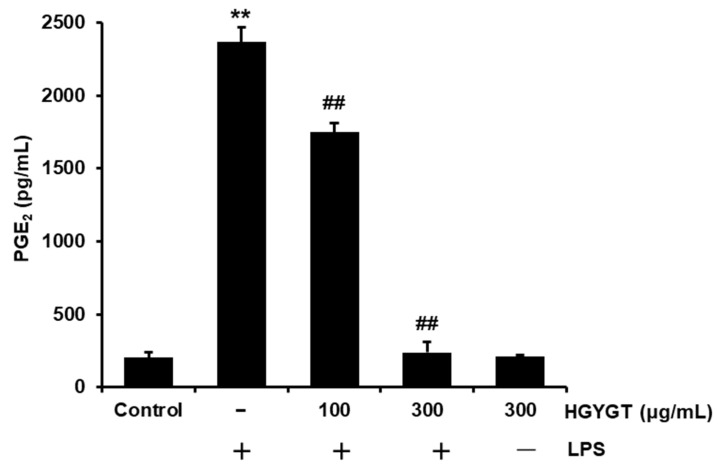
Effects of HGYGT on prostaglandin E2 (PGE2) production by LPS in RAW 264.7 cells. The cells at a concentration of 5 × 10^5^ cells/mL were treated with different concentrations (100 and 300 μg/mL) of HGYGT for 1 h, followed by induction with 1 µg/mL of LPS, and these cells were then incubated for 20 h. Control cells were incubated with vehicle alone. The concentrations of PGE2 in the culture supernatant were recorded. Data are presented as mean ± S.D. of three replicates for each sample. Statistical analysis was performed using one-way ANOVA, followed by Tukey’s HSD or Dunnett T3 test. ** *p* < 0.01 significant compared with vehicle-treated control. ^##^ *p* < 0.01 significant compared with LPS-only group.

The effect of HGYGT on LPS-induced PGE2 production was measured. Compared to the control, treatment with LPS significantly increased PGE2 production (*p* < 0.01, Figure 3). However, treatment with HGYGT (100 and 300 μg/mL) significantly inhibited LPS-induced PGE2 production by 26.1% and 90%, respectively (*p* < 0.01, Figure 3).

### 3.4. Effects of HGYGT on LPS-Stimulated iNOS, COX-1, and COX-2 Protein Expression

**Figure 4:**
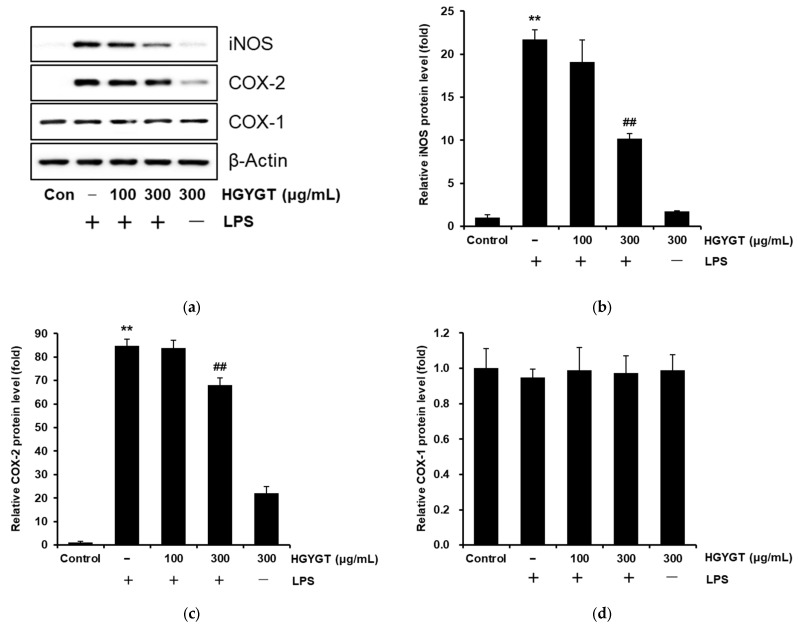
Inhibitory effects of HGYGT on LPS-induced expression of inducible nitric oxide synthase (iNOS), cyclooxygenase (COX)-2, and COX-1 (**a**). RAW 264.7 cells at a concentration of 5 × 10^5^ cells/mL were treated with different concentrations (100 and 300 μg/mL) of HGYGT for 1 h, followed by induction with 1 µg/mL of LPS, and the cells were then incubated for 20 h. Control cells were incubated with vehicle alone. The protein levels of COX-1, COX-2, and iNOS were examined by Western blot analysis. β-Actin was applied as a control. β-Actin versus COX-1, COX-2, and iNOS were recorded via densitometry (**b**–**d**). Data are presented as mean ± S.D. of three replicates for each sample. Statistical analysis was performed using one-way ANOVA, followed by Tukey’s HSD or Dunnett T3 test. ** *p* < 0.01 significant compared with vehicle-treated control. **^##^** *p* < 0.01 significant compared with LPS-only group.

Western blotting was performed to examine whether the inhibitory effects of HGYGT were associated with the expression of iNOS, COX-1, and COX-2. Levels of iNOS and COX-2 protein were highly upregulated in response to LPS (*p* < 0.01, Figure 4a–c), whereas treatment with HGYGT (300 μg/mL) significantly inhibited levels of iNOS by 53% and COX-2 by 19.9% (*p* < 0.01, Figure 4b,c). However, COX-1 expression did not show any significant difference between the LPS and HGYGT treatments (Figure 4d).

### 3.5. Effects of HGYGT on LPS-Induced IL-1β, IL-6, and TNF-α Production

**Figure 5:**
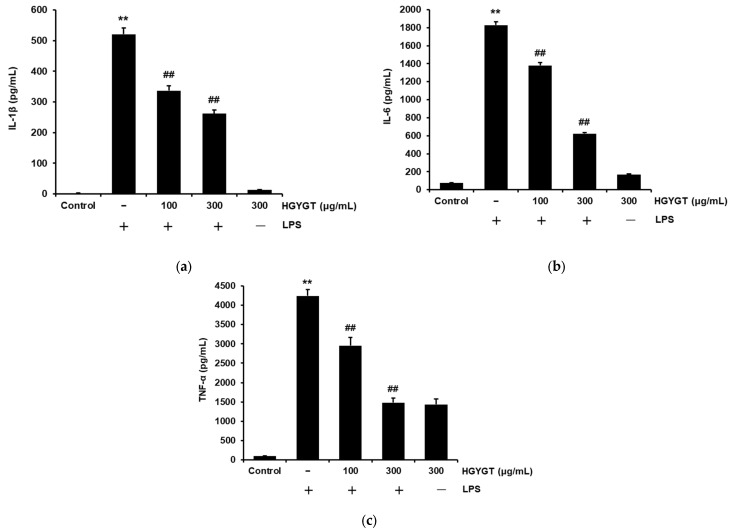
Inhibitory effects of HGYGT on LPS-induced production of interleukin 1-beta (IL-1β), interleukin-6 (IL-6), and tumor necrosis factor-alpha (TNF-α) (**a**–**c**) in RAW 264.7 cells. The cells (5 × 10^5^ cells/mL) were treated with HGYGT (100 and 300 μg/mL) for 1 h, followed by induction with 1 µg/mL of LPS, and the cells were then incubated for 20 h. Control cells were incubated with vehicle alone. The levels of pro-inflammatory cytokines were measured by ELISA. Data are presented as mean ± S.D. of three replicates for each sample. Statistical analysis was performed using one-way ANOVA, followed by Tukey’s HSD or Dunnett T3 test. ** *p* < 0.01 significant compared with control. ^##^ *p* < 0.01 significant compared with LPS-only group.

We investigated whether HGYGT exhibited anti-inflammatory efficacy by inhibiting the production of inflammatory cytokines such as IL-1β, IL-6, and TNF-α in LPS-activated RAW 264.7 cells. When compared to the control, treatment with LPS significantly increased the production of IL-β, IL-6, and TNF-α (*p* < 0.01, Figure 5). Treatment with HGYGT (100 and 300 μg/mL) significantly inhibited LPS-induced IL-1β (35.3%, 49.7%), IL-6 (24.5%, 66%), and TNF-α (30.3%, 65%) production (*p* < 0.01, Figure 5).

### 3.6. Effects of HGYGT on LPS-Stimulated Activation of NF-κB

**Figure 6:**
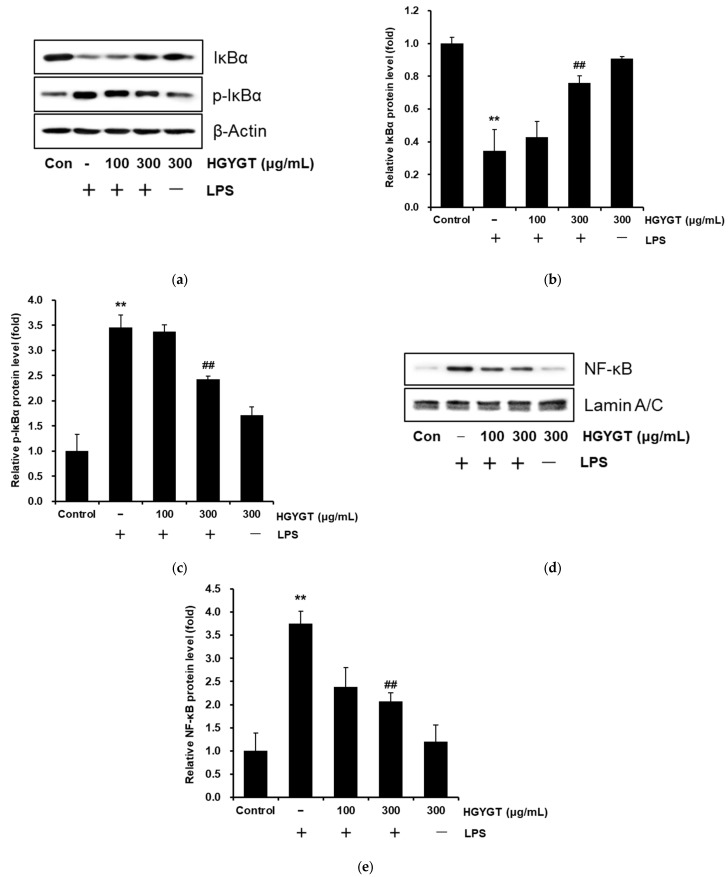
Inhibitory effects of HGYGT on LPS-induced activation of inhibitor of κB alpha (IκBα) (**a**), phosphorylated (p)-IκBα (**a**), and nuclear factor kappa-light-chain enhancer of activated B cells (NF-κB) (p65) (**d**). RAW 264.7 cells at a concentration of 5 × 10^5^ cells/mL were treated with HGYGT (100 and 300 μg/mL) for 1 h, then with LPS (1 μg/mL) for 15 min. Control cells were treated with vehicle only. Nuclear extracts were prepared as described in the Section 2. Western blot analysis was performed to determine the protein levels of IκBα, p-IκBα, and NF-κB (p65 subunit). IκBα and p-IκBα vs. β-actin and NF-κB (p65) vs. Lamin A/C ratios were measured by densitometry (**b**,**c**,**e**). Data are presented as mean ± S.D. of three replicates for each sample. Statistical analysis was performed using one-way ANOVA, followed by Tukey’s HSD or Dunnett T3 test. ** *p* < 0.01 significant compared with vehicle-treated control. ^##^ *p* < 0.01 significant compared with LPS-only group.

To comprehend whether inhibition of the nuclear translocation of NF-κB (p65) by HGYGT was due to the suppression of p-IκBα and the upregulation of IκBα, a Western blot analysis was conducted to evaluate the levels of IκBα, p-IκBα, and NF-κB (p65). Treatment with LPS increased the level of p-IκBα at 15 min, and HGYGT (300 μg/mL) significantly blocked the LPS-stimulated increase by 29.8% (*p* < 0.01, Figure 6a,c). But, the levels of IκBα were reversed by 202% (Figure 6a,b). In addition, NF-κB (p65) was activated at 15 min after treatment with LPS, and the LPS-stimulated NF-κB activation was significantly inhibited by 44.9% with HGYGT (300 μg/mL) (*p* < 0.01, Figure 6d,e).

### 3.7. Inhibitory Effects of HGYGT on LPS-Stimulated Phosphorylation of MAPKs

**Figure 7:**
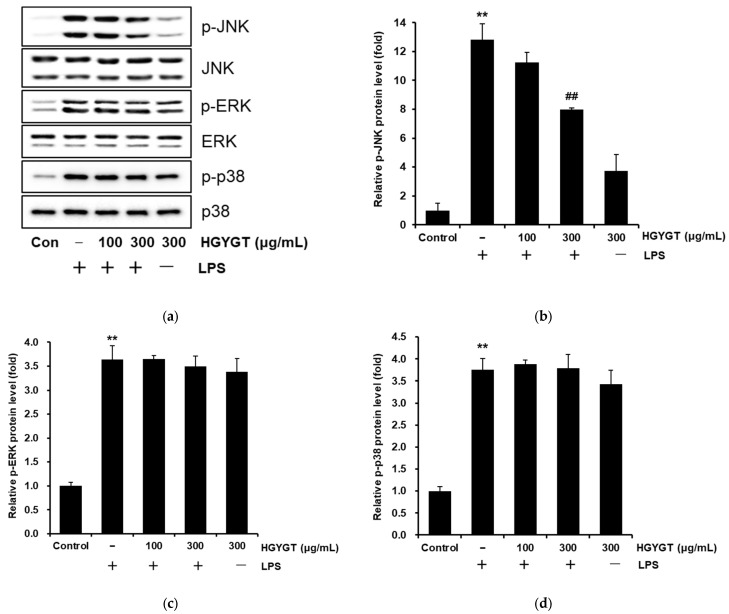
Inhibitory effects of HGYGT on phosphorylation of mitogen-activated protein kinases (MAPKs) (i.e., c-Jun N-terminal kinase (JNK), extracellular signal-regulated kinase (ERK), and p38) in LPS-stimulated RAW 264.7 cells (**a**). The cells were treated with the indicated concentrations (100 and 300 μg/mL) of HGYGT for 1 h, followed by induction with 1 μg/mL LPS for 15 min. Western blot analysis was conducted using anti-phosphokinase antibodies for cell extract analysis. Detection of non-phosphorylated kinases by Western blot analysis was performed with the protein control. The blots shown are the best of three blots. The phosphorylation of MAPK intensity was determined by densitometry (**b**–**d**). Data are presented as mean ± S.D. of three replicates for each sample. Statistical analysis was performed using one-way ANOVA, followed by Tukey’s HSD or Dunnett T3 test. ** *p* < 0.01 significant compared with vehicle-treated control. ^##^ *p* < 0.01 significant compared with LPS-only group.

To evaluate the molecular target of HGYGT in the upstream signaling pathway, we investigated the effects of HGYGT on the LPS-stimulated phosphorylation of JNK, ERK1/2, and p38 MAPKs in RAW 264.7 cells. LPS treatment significantly increased the phosphorylation of JNK, ERK1/2, and p38 MAPKs (*p* < 0.01, Figure 7). However, HGYGT treatment (300 μg/mL) significantly decreased the phosphorylation of JNK by 37.7% (*p* < 0.01, Figure 7a,b) but did not affect the phosphorylation of ERK1/2 and p38 (Figure 7a,c,d).

### 3.8. Effects of HGYGT on CA-Induced Paw Edema and Expression of iNOS Protein by CA in the Paw Tissues

**Figure 8:**
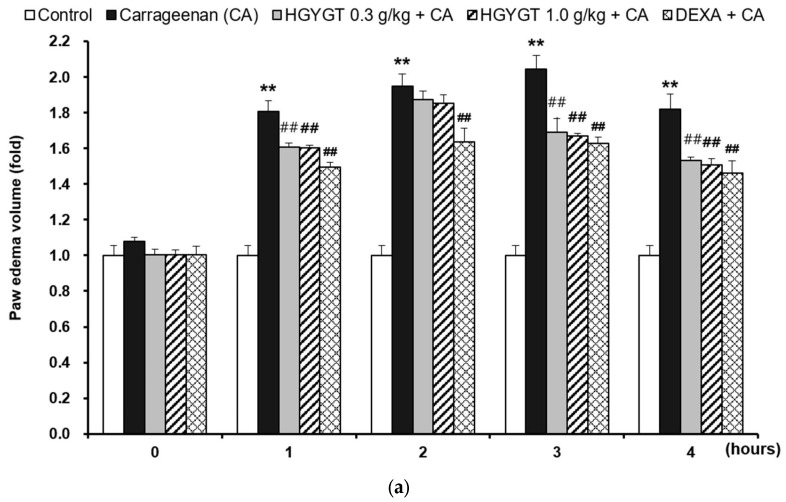

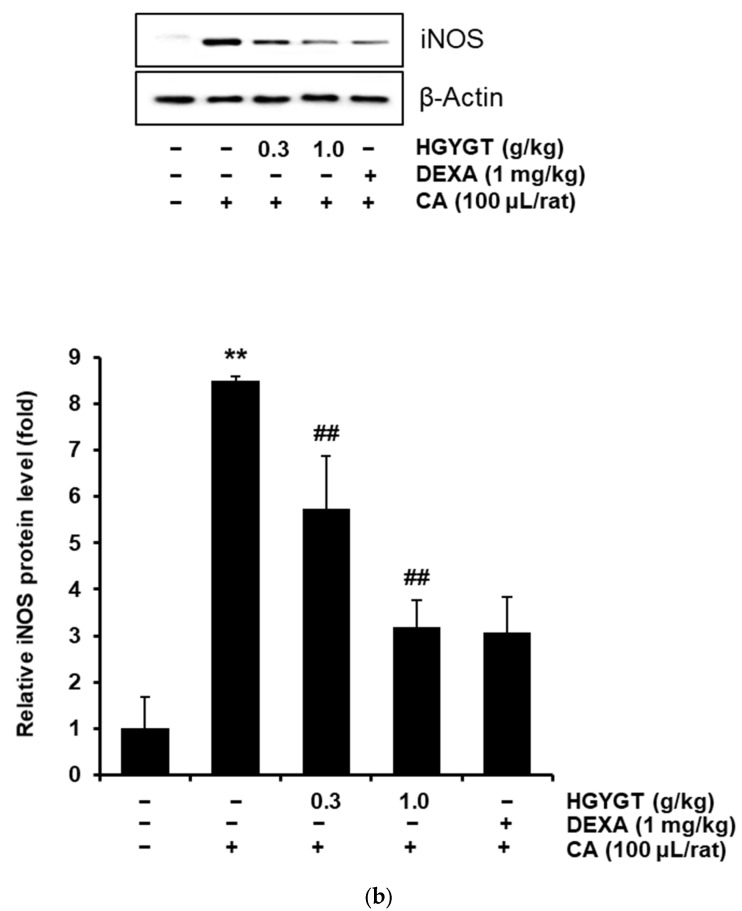
Effects of HGYGT on carrageenan (CA)-induced paw edema (**a**) and expression of iNOS (**b**) by CA in the paw tissues. Rats were orally pretreated with dexamethasone (DEXA, 1 mg/kg, p.o., 3 days) or HGYGT (0.3, 1.0 g/kg, p.o., 3 days) and subcutaneously injected with 1% CA (100 μL/rat, dissolved in sterilized saline). (**a**) The volume of paw swelling was recorded 0–4 h after CA injection. (**b**) The paw tissue samples prepared from rats at 4 h after CA injection were subjected to separation of protein. The expression of iNOS protein was immunoblotted in the tissue samples. Equal protein loading among samples was verified by β-actin immunoblotting. Data are presented as mean ± S.D. of five animals. Statistical analysis was performed using one-way ANOVA, followed by Tukey’s HSD or Dunnett T3 test. ** *p* < 0.01 significant compared with control. ^##^ *p* < 0.01 significant compared with CA alone.

In this study, treatment with CA resulted in a significant increase in paw swelling compared to the control group (*p* < 0.01, Figure 8a). However, treatment with DEXA (positive control, 1 mg/kg/day per os) significantly reduced edema at all time points, with reductions of 17.4% at 1h, 16% at 2h, 19.6% at 3h, and 19.6% at 4h (*p* < 0.01, Figure 8a). Similarly, treatment with HGYGT (0.3 and 1.0 g/kg/day, per os, 3 days) significantly decreased the paw edema volumes, except at 2 h after CA injection. At 1h, 3h, and 4h post-injection, HGYGT 0.3 g/kg reduced the swelling by 11.2%, 17.1%, and 15.7%, respectively, while HGYGT **1.0 g/kg** led to reductions of **11.3%, 18.3%, and 17.0%** (*p* < 0.01, Figure 8a). In paw tissues, treatment with CA resulted in a significantly increased expression of iNOS protein relative to that in the control group (*p* < 0.01; Figure 8b). However, treatment with DEXA significantly decreased iNOS protein expression by 63.8% (*p* < 0.01; Figure 8b). Treatment with HGYGT (0.3 and 1.0 g/kg) also resulted in significantly decreased expression of iNOS protein by 32.3% and 62.6% (*p* < 0.01, Figure 8b).

### 3.9. Effects of HGYGT on Plasma Levels of ALT and AST

**Figure 9:**
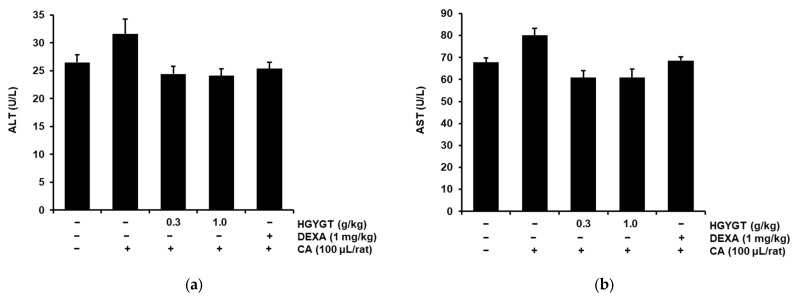
Effects of HGYGT on plasma levels of alanine aminotransferase (ALT) and aspartate aminotransferase (AST). The blood levels of ALT (**a**) and AST (**b**) were measured. Data are recorded as mean ± S.D. Statistical analysis was performed using one-way ANOVA, followed by Tukey’s HSD or Dunnett T3 test. ALT, alanine aminotransferase; AST, aspartate aminotransferase.

The effects of HGYGT on the plasma levels of ALT and AST in rats were examined. CA treatment does not induce hepatotoxicity. In addition, the levels of ALT and AST were not significantly different between the HGYGT-treated (0.3 and 1.0 g/kg) and control groups. Finally, the DEXA group showed similar levels of ALT and AST as the control group (Figure 9).

These data indicate that no hepatotoxicity was induced by HGYGT at different doses and periods in this study.

### 3.10. Effects of HGYGT on CA-Induced Paw Edema Through Histological Examination

**Figure 10:**
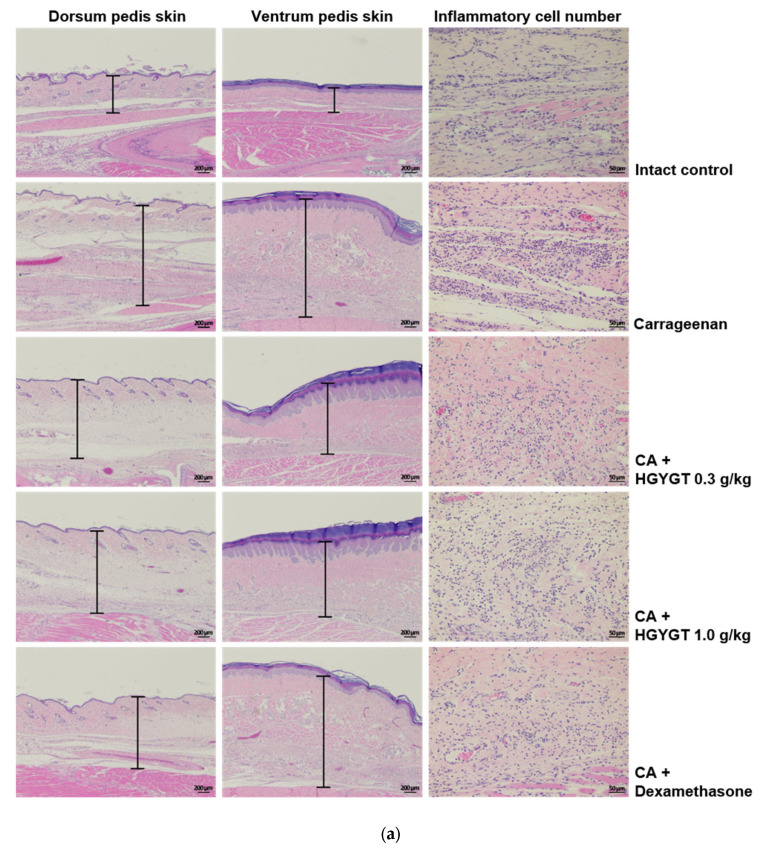

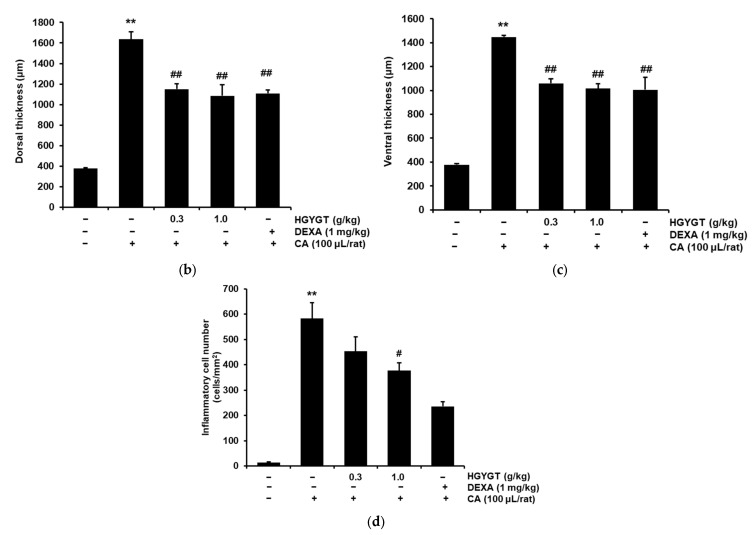
Histological images of *dorsum* and *ventrum pedis* skin and changes on the histomorphometrical analysis of paw skins. *Dorsum* and *ventrum pedis* tissue sections from the control, CA, CA + 0.3 g/kg HGYGT, CA + 1.0 g/kg HGYGT, and CA + DEXA rat groups were stained with hematoxylin and eosin (H and E) for histopathological analysis. Bars indicate skin thickness (**a**). *Dorsal* and *ventral* thickness (**b**,**c**) and inflammatory cell number were measured (**d**). Statistical analysis was performed using one-way ANOVA, followed by Tukey’s HSD or Dunnett T3 test. ** *p* < 0.01 significant compared with control. ^#^ *p* < 0.05, ^##^ *p* < 0.01 significant compared with CA alone. HGYGT, *hyeonggaeyeongyo-tang*; CA, carrageenan; DEXA, dexamethasone.

Paw edema induced by CA was measured over a 4 h period, after which the rats were sacrificed to assess skin thickness and inflammatory cell infiltration in both the dorsum pedis and ventrum pedis (Figure 10). Compared to the control group, the dorsum and ventrum pedis skins were significantly thicker in the CA group (*p* < 0.01, Figure 10b,c). However, treatment with HGYGT (0.3 and 1.0 g/kg) significantly reduced the skin thickness compared to the CA group. Specifically, in the dorsum pedis, HGYGT 0.3 g/kg reduced thickness by 29.7%, while HGYGT 1.0 g/kg led to a 33.6% reduction (*p* < 0.01). DEXA (1 mg/kg) also significantly reduced dorsal skin thickness by 32.3% (*p* < 0.01, Figure 10b). Similarly, in the ventrum pedis, HGYGT 0.3 g/kg reduced thickness by 26.8%, and HGYGT 1.0 g/kg reduced it by 29.8% (*p* < 0.01). DEXA treatment resulted in a 30.5% reduction (*p* < 0.01, Figure 10c). Furthermore, compared to the control group, the CA group showed a significant increase in infiltrated inflammatory cells in the ventrum pedis (*p* < 0.01). While HGYGT 0.3 g/kg did not significantly reduce inflammatory cell infiltration, HGYGT 1.0 g/kg led to a 35.2% reduction (*p* < 0.05), and DEXA treatment resulted in a 59.6% reduction (*p* < 0.01, Figure 10d).

## 4. Discussion

One of the primary challenges in herbal medicine research is the variability of raw herbal materials, which often lacks the standardization seen in conventional pharmaceuticals. Factors, such as geographical origin, cultivation method, and harvest conditions, can significantly influence the chemical composition of herbal extracts, leading to inconsistent experimental outcomes. To address this issue, UPLC was employed prior to conducting anti-inflammatory experiments in this study, allowing for precise quality control and the standardization of the HGYGT extracts. Particularly, 14 representative components, forsythoside B, platycodin D, ligustrazine hydrochloride, paeoniflorin, paeonol, catalpol, berberine, decursinol, glycyrrhizic acid, isoimperatorin, luteolin-7-glucopyranoside, saikosaponin D, peucedanol, and geniposide, were analyzed and standardized using UPLC. By ensuring the consistency and reproducibility of the HGYGT extract, this approach not only mitigates the inherent variability of herbal medicines but also strengthens the scientific rigor and reliability of the present study’s findings.

NO is produced via l-arginine oxidation by NOS with L-citrulline [4]. Among NOS, iNOS is expressed by specific stimuli, such as LPS, TNF-α, and IL-1 in macrophages, hepatocytes, and stellate cells, and produces large amounts of NO for a long period of time [20]. Continuous high-concentration NO production can cause tissue damage due to damage to host cells and an induction of inflammation [21]. In this study, HGYGT significantly inhibited the NO production induced by LPS treatment at concentrations of 100 and 300 μg/mL. In addition, when cell viability was evaluated to assess whether this was due to cytotoxicity, it did not show significant cytotoxicity compared to the LPS-only treatment group, implying that HGYGT itself inhibited NO production mediated by the inflammatory response. As a result of evaluating iNOS expression, the iNOS expression increased by LPS was significantly reduced at a concentration of 300 μg/mL of HGYGT. These results indicated that HGYGT inhibited iNOS expression without cytotoxicity, thereby inhibiting NO production.

Additionally, COX-2 is activated during inflammatory responses to produce PGE2, which contributes to tumorigenesis by inducing cell division, cancer metastasis, angiogenesis, and inhibiting apoptosis [22]. In this study, HGYGT significantly inhibited PGE2 production in LPS-stimulated RAW 264.7 cells at 100 and 300 μg/mL, and COX-2 expression was also significantly inhibited at 300 μg/mL. COX-2, which is induced by inflammatory responses, was inhibited. However, COX-1, which is constantly expressed and maintains normal physiological functions, was not reduced by HGYGT or LPS + HGYGT treatment. These results suggested that HGYGT may have therapeutic effects in the treatment of pathogenic heat and pain. Furthermore, IL-1 is mainly secreted by macrophages, monocytes, fibroblasts, and endothelial cells, and there are two major forms of IL-1, IL-1α and IL-1β. Although their sequence homology is only 25%, both bind to IL-1R and exhibit similar biological functions. They are involved in fever, immune and inflammatory responses, injury promotion, wound healing, and scar formation. Unlike IL-1β, IL-1α can translocate to the nucleus and act as a transcriptional activator that helps the inflammatory and fibrotic pathways associated with IL-1β [23,24]. IL-6 is secreted by various cells, such as fibroblasts, keratinocytes, mesangial cells, vascular endothelial cells, mast cells, macrophages, dendritic cells, T cells, and B cells, and a dysregulation of IL-6 can induce inflammation, autoimmune diseases, and tumors [25]. TNF-α is a pro-inflammatory cytokine that is critically involved in autoimmune diseases. It was initially recognized as an endogenous factor that induces necrosis of solid tumors. However, its importance as a pro-inflammatory cytokine involved in immune pathogenesis has gradually increased. In the liver, TNF-α is known to induce the apoptosis and necroptosis of hepatocytes, hepatitis, autoimmunity, and progression to hepatocellular carcinoma [26]. In this study, LPS significantly increased the production of IL-1β, IL-6, and TNF-α, and HGYGT (100 and 300 μg/mL) significantly reduced the increased IL-1β, IL-6, and TNF-α. Therefore, it was confirmed that HGYGT exhibits anti-inflammatory efficacy by inhibiting the production of the inflammatory cytokines IL-1β, IL-6, and TNF-α.

NF-κB is a transcription factor that regulates the expression of genes involved in immune system regulation and inflammatory response regulation. NF-κB was first discovered by Ranjan Sen and David Baltimore in 1986, and as its name suggests, it binds to the immunoglobulin κ light chain enhancer of activated B cells and regulates the induction of various target genes involved in cell cycle, differentiation, activation, and apoptosis. NF-κB has five NF-κB subtypes: RelA (p65), RelB, c-Rel, p105, and p100. In particular, p65 and p50 are involved in the canonical activation of the NF-κB signaling pathway and regulating innate immunity. However, excessive and uncontrolled activation of NF-κB causes the development and progression of inflammatory lesions [27]. In addition, NF-κB binds to IκB and exists in an inactive state in the cytoplasm, but when cells are stimulated externally, NF-κB is separated through the phosphorylation process of IκB. The separated NF-κB passes through the nuclear membrane and moves into the nucleus to promote the transcription of various inflammation-related genes [28]. Therefore, the regulation of NF-κB activation and movement into the nucleus is important for anti-inflammatory research. In this study, the expression of IκBα was decreased in the RAW 264.7 cells activated with LPS, whereas the expressions of p-IκBα and NF-κB were increased. However, the expression of IκBα was significantly increased by HGYGT (300 μg/mL), and the expressions of p-IκBα and NF-κB were significantly decreased at 300 μg/mL. Collectively, these data indicate that the inhibitory activities of HGYGT against the activation of NF-κB show correlation with the reduction of induction of iNOS, COX-2, and pro-inflammatory cytokines.

In addition, MAPKs consist of the JNK, ERK1/2, and p38 signaling pathways and are involved in the activation of inflammation-related transcription factors, including NF-κB [29]. Many studies have demonstrated that MAPKs play important roles in inflammation and cancer development [30]. In this study, to evaluate whether HGYGT’s inhibition of inflammatory mediator production and NF-κB activation was due to the regulation of MAPKs signaling, the expressions of JNK, ERK1/2, and p38 proteins were examined through immunoblot analysis. LPS treatment alone significantly increased the phosphorylation of JNK, ERK1/2, and p38. HGYGT significantly reduced the phosphorylation of JNK by LPS at 300 μg/mL, but did not affect the phosphorylation of ERK1/2 and p38. These results suggest that HGYGT exerts its anti-inflammatory effects through the JNK signaling pathway.

This induces an inflammatory response [31,32,33]. Accordingly, CA-induced animal models of acute paw edema have been widely used to determine the anti-inflammatory effects of various drugs [34,35]. Histopathologically, loosening of the connective tissue and inflammatory cell infiltration have been observed around CA-treated sites [36,37]. In the present study, a prominent increase in the number of infiltrating inflammatory cells in the *ventrum* tissue was observed after treatment with CA. However, this increase in inflammatory cells was significantly reduced by treatment with 1.0 g/kg HGYGT and DEXA (1 mg/kg, an anti-inflammatory drug). In skin thickness experiments, the *dorsum* and *ventrum pedis* skins were significantly thicker in the CA group. However, compared to the CA group, the *dorsum* and *ventrum pedis* skins were significantly thinner in the 0.3 and 1.0 g/kg HGYGT and DEXA (1 mg/kg). These results indicate that HGYGT has anti-inflammatory activity.

Chromatographic analysis revealed that the primary markers of HGYGT were forsythoside B, platycodin D, ligustrazine hydrochloride, paeoniflorin, paeonol, catalpol, berberine, decursinol, glycyrrhizic acid, isoimperatorin, geniposide, luteolin-7-glucopyranoside, saikosaponin D, and peucedanol. Forsythoside B attenuates neuroinflammation and neuronal apoptosis by inhibiting the NF-κB and p38-MAPK signaling pathways through Nrf2 activation after spinal cord injury [38]. Platycodin D relieves rheumatoid arthritis by inducing mitochondrial apoptosis and inhibiting the hedgehog signaling pathway [39]. Ligustrazine hydrochloride protects the vascular endothelium in patients undergoing cardiopulmonary bypass by reducing excessive coagulation and inflammatory responses [40]. Paeoniflorin has anti-inflammatory and immunoregulatory effects [41]. Paeonol alleviates ulcerative colitis in mice by promoting short-chain fatty acid production in Clostridium butyricum [42]. Catalpol suppresses pro-inflammatory mediators, such as NO, IL-6, and TNF-α, in LPS-stimulated BV2 microglial cells through NF-κB pathway inhibition [43]. Berberine reduces inflammatory cytokine production (IL-1β, IL-6, and TNF-α) and modulates both the NF-κB and MAPK signaling pathways [44]. Decursinol exhibits antitumor, anti-inflammatory, and antioxidant effects [45]. Glycyrrhizic acid mitigates LPS-induced acute lung injury by regulating the ACE2 and caveolin-1 signaling pathways, contributing to inflammatory regulation [46]. Isoimperatorin alleviates LPS-induced periodontitis by downregulating the ERK1/2 and NF-κB pathways [47]. Geniposide suppresses psoriatic skin inflammation by inhibiting the TLR4/MyD88/NF-κB p65 signaling pathway and MMP9 [48]. Luteolin-7-glucopyranoside exhibits antioxidant and anti-inflammatory properties by modulating the JAK1/STAT6/SOCS1 pathway and alleviating inflammatory diseases, such as colitis [49]. Saikosaponin D inhibits the NF-κB pathway and reduces iNOS, COX-2, and pro-inflammatory cytokine expression [50]. Peucedanol derivatives, namely peucedanol 7-O-β-D-apiofuranosyl (1→6)-β-glucopyranoside, and peucedanol 7-O-β-D-glucopyranoside, exhibit anti-inflammatory activity by inhibiting NO production [51].

Therefore, these results suggest that the anti-inflammatory activities of complex herbal medications, such as nose-type HGYGT, may be superior to those of a single compound, owing to the synergistic effects of the individual ingredients.

This study has several limitations. First, the CA-induced paw edema model employed in this study did not directly correspond to nasal conditions such as rhinitis or sinusitis, thereby limiting the direct clinical applicability of the findings. Second, this study focused exclusively on acute inflammation models, leaving the effects of HGYGT in chronic inflammatory conditions unexplored. Third, although this study confirmed the anti-inflammatory effects of HGYGT in cellular and animal models, this study did not include clinical trials, nor did it assess HGYGT’s comparative efficacy against existing anti-inflammatory treatments or its safety at higher concentrations. Additionally, although 14 marker compounds were identified in HGYGT, this study did not determine which specific components contribute most significantly to its anti-inflammatory effects. Given that herbal formulations often rely on complex synergistic interactions, identifying key bioactive compounds could help optimize the formulation for improved efficacy. Furthermore, while AST and ALT levels in this study indicated no immediate hepatotoxicity, the long-term effects on liver and kidney function were not assessed. Evaluating potential toxicity with prolonged use is essential to ensure its long-term safety.

Despite these limitations, this study provides significant academic contributions and novel insights into the therapeutic potential of HGYGT. This is the first study to demonstrate that HGYGT selectively inhibits COX-2 without affecting COX-1, thereby distinguishing itself from conventional NSAIDs by achieving effective anti-inflammatory effects while minimizing the associated side effects. Moreover, this study elucidated the specific mechanisms underlying the anti-inflammatory actions of HGYGT, including the suppression of PGE2 production and selective inhibition of the JNK pathway within the MAPKs signaling cascade. Through the integration of histological analyses, this study further substantiated the anti-inflammatory efficacy of HGYGT at both the cellular and tissue levels. Additionally, this study highlights the clinical applicability of HGYGT by targeting NF-κB, a pivotal transcription factor in inflammatory regulation, thereby providing robust evidence for its use in the treatment of inflammatory diseases.

To overcome these limitations and enhance the translational relevance of the findings, future research should incorporate animal models that more accurately represent nasal conditions, such as rhinitis or sinusitis. Furthermore, the long-term efficacy of HGYGT should be evaluated in studies focusing on chronic inflammatory models. Most importantly, well-designed clinical trials should be conducted to confirm the therapeutic potential of HGYGT in human patients to ensure its clinical safety and efficacy in real-world applications. In addition, direct comparisons with existing anti-inflammatory treatments, such as NSAIDs and corticosteroids, are necessary to assess HGYGT’s relative therapeutic advantages. Investigating its benefits over standard therapies, particularly its selective COX-2 inhibition and reduced side-effect profile, will help determine its viability as an alternative or complementary treatment for inflammatory diseases. Further studies should also explore the potential cytotoxicity of HGYGT at higher concentrations to establish its dose-dependent safety profile and long-term therapeutic viability. Moreover, since our UPLC analysis identified 14 marker compounds in HGYGT, future research should analyze the individual and combined effects of these bioactive compounds. Understanding their specific contributions and potential synergistic interactions could refine the formulation to maximize its therapeutic efficacy. Lastly, given that this study only assessed acute toxicity through AST and ALT levels, future research should investigate the long-term safety of HGYGT, particularly its effects on liver and kidney function with prolonged use. Chronic toxicity studies in animal models, followed by clinical evaluations, will be crucial to ensure its safety for extended therapeutic applications.

## 5. Conclusions

This study clearly demonstrated that HGYGT exerts significant anti-inflammatory effects by inhibiting the JNK and NF-κB signaling pathways in LPS-induced RAW 264.7 cells, thereby reducing the production of inflammatory mediators such as PGE2, NO, and pro-inflammatory cytokines. In addition, in a CA-induced rat paw edema model, HGYGT exhibited anti-edema effects at a dose of 1.0 g/kg by suppressing acute edematous inflammation, as evidenced by a reduction in skin thickness and inflammatory cell infiltration. Notably, unlike previous studies, this study revealed that HGYGT selectively inhibits COX-2 without affecting COX-1 and specifically suppresses the JNK signaling pathway within the MAPKs pathway. Furthermore, iNOS inhibition and histological analyses in a CA-induced rat paw edema model comprehensively demonstrated the in vivo anti-inflammatory effects of HGYGT. Our findings provide preclinical evidence supporting the traditional use of HGYGT in treating inflammatory diseases. While these results demonstrate its anti-inflammatory properties in cellular and animal models, further clinical studies are necessary to confirm its therapeutic potential in humans.

## Figures and Tables

**Table 1:** Amount and composition of *hyeonggaeyeongyo-tang* (HGYGT).

Scientific Name	Dose (g)
*Schizonepeta tenuifolia* Briq	1.875 g
*Bupleurum falcatum* L.	1.875 g
*Cnidium officinale* Makino	1.875 g
*Angelica gigas* Nakai	1.875 g
*Rehmannia glutinosa* (Gaertn.) DC	1.875 g
*Paeonia lactiflora* Pall	1.875 g
*A. dahurica* (Hoffm.) Benth. & Hook.f. ex Franch. and Sav	1.875 g
*Saposhnikovia divaricata* (Turcz.) Schischk	1.875 g
*Mentha arvensis* L.	1.875 g
*Gardenia jasminoides* J. Ellis	1.875 g
*Scutellaria baicalensis* Georgi	1.875 g
*Platycodon grandiflorum* (Jacq.) A. DC	1.875 g
*Forsythia suspensa* (Thunb.) Vahl	1.875 g
*Glycyrrhiza uralensis* Fisch	1.125 g

**Table 2:** Gradient profile for the 14 compounds and HGYGT analysis of ultra-performance liquid chromatography (UPLC).

Time (min)	0.1% FA/Water (%)	0.1% FA/Acetonitrile (%)	Flow Rate (mL/min)
0	98	2	0.40
1.0	98	2	0.40
2.0	90	10	0.40
5.0	65	35	0.40
6.0	50	50	0.40
7.0	50	50	0.40
9.0	30	70	0.40
13.0	10	90	0.40
14.0	2	98	0.40
15.0	98	2	0.40
16.0	98	2	0.40

UPLC, ultra-performance liquid chromatography; FA, formic acid. The 14 compounds that were identified: Geniposide, Glycyrrhizic acid, Isoimperatorin, Ligustrazine Hydrochloride, Paeonol, Forsythoside B, Platycodin D, Luteolin-7-glucopyranoside, Saikosaponin D, Peucedanol, catalpol, Decursinol, Paeoniflorin, Berberine.

**Table 3:** Contents of 14 marker compounds in HGYGT by UPLC.

Compound	Content (μg/g)
Forsythoside B	93.842 ± 0.649
Platycodin D	39.998 ± 1.363
Ligustrazine Hydrochloride	5.687 ± 0.207
Paeoniflorin	42.873 ± 2.632
Paeonol	0.417 ± 0.044
Catalpol	11.057 ± 0.645
Berberine	2.660 ± 0.086
Decursinol	2.977 ± 0.280
Glycyrrhizic acid	21.279 ± 1.244
Isoimperatorin	0.369 ± 0.021
Geniposide	71.665 ± 4.486
Luteolin-7-glucopyranoside	10.437 ± 0.428
Saikosaponin D	2.054 ± 0.135
Peucedanol	20.507 ± 0.375

Data are presented as mean ± S.D. of three replicates for each sample.

## Data Availability

The data supporting this study are available upon request from the corresponding author. The dataset is not publicly available due to the nature of the research.

## Abbreviations

The following abbreviations are used in this manuscript:

ALT	Alanine Aminotransferase
AST	Aspartate Aminotransferase
BCA	Bicinchoninic Acid
CA	Carrageenan
COX	Cyclooxygenase
DEXA	Dexamethasone
DMEM	Dulbecco’s Modified Eagle’s Medium
ECL	Enhanced Chemiluminescence
ELISA	Enzyme-Linked Immunosorbent Assay
ERK	Extracellular Signal-Regulated Kinase
FA	Formic acid
FBS;	Fetal Bovine Serum
HGYGT	*hyeonggaeyeongyo-tang*
IL-1β	Interleukin-1 beta
IL-6	Interleukin-6
iNOS	Inducible Nitric Oxide Synthase
IκBα	Inhibitor of κB alpha
JNK	c-Jun N-terminal Kinase
LPS	Lipopolysaccharide
MAPKs	Mitogen-Activated Protein Kinases
MTT	3-(4,5-Dimethylthiazol-2-yl)-2,5-diphenyltetrazolium bromide
NC membrane	Nitrocellulose Membrane
NF-κB	Nuclear Factor kappa-Light-Chain-Enhancer of Activated B cells
NO	Nitric Oxide
PBS	Phosphate-Buffered Saline
PDA	Photodiode Array
p-ERK	Phosphorylated Extracellular Signal-Regulated Kinase
PGE2	Prostaglandin E2
p-IκBα	Phosphorylated Inhibitor of κB alpha
p-JNK	Phosphorylated c-Jun N-Terminal Kinase
p-p38	Phosphorylated p38 MAPK
RAW	Research Applications of Wistar Institute
SDS-PAGE:	Sodium Dodecyl Sulfate–Polyacrylamide Gel Electrophoresis
TCMTNF-α	Traditional Chinese medicineTumor Necrosis Factor-alpha
Tukey’s HSD test	Tukey’s Honestly Significant Difference test
UPLC	Ultra-Performance Liquid Chromatography
